# A systematic methodology review of fluorescence-guided cancer surgery to inform the development of a core master protocol and outcome set

**DOI:** 10.1186/s12885-024-12386-4

**Published:** 2024-06-07

**Authors:** Abigail E. Vallance, The CLEARER study collaborative*, Daniel Elson, Stefano Giuliani, Kenneth Rankin, Graeme Stasiuk, Myles Smith, Daniel Leff, Vinidh Paleri, Angus McNair, Erum Ahmad, Hashim Ahmed, Antony Antypas, Amir Anuar, Alice Appleton, Cara Beattie, Disha Bhadbury, Rhiannon Brignall, Claudia Burton, Ollie Burton, Janice Chow, Howard Chu, Kelly Chu, Brian Cunningham, Elizabeth Daly, Noor Dhakal, Michael Douek, Ben Doughty, Kaylem Feeney, Alex Fleet, Hayley Fowler, Michael Fu, Mark Galea, Hannah Glatzel, Esther Goh, Hannah Grimes, Mei-Yin Gruber, Natalia Hackett, Mark Hanson, Jessica Helm, George Higginbottham, Rayyan Islam, Alisha Jaffer, Marwa Jama, Rama Jha, Jade Kabbani, Jamil Kabbani, Ayesha Kahn, Jessica Kennett, Ariella Levene, Ethan Losty, Andie Lun, Krzysztof Macierzanka, Fahad Mahmood, Jed Maliyil, Emily-Jane Mitchell, Intisar Mohamed, Ali Mohammed, Marco Mund, James Odedra, Olufemi Olatigbe, Maeve O’Neill, Daniel-Clement Osei-Bordom, Ariadni Papadopoulou, Manal Patel, Arnie Purushotham, Fang Fang Quek, Euan Ramsay, Luke James Roberts, Augustus Rottenberg, Elizabeth Ryan Harper, Lucy Scales, Preeyan Shah, Chloe Short, Keng Siang Lee, Eleanor Smyth, Ollie Squires, Aiswarya Sukumar, Harsha Thangavijayan, Arun Thirunavukarasu, Dalia Thomas, Carrie Thorpe, Alexandra Uren, Jayant Vaidya, Florence Wallace, Nora Wangari Murage, Mary Xie Lee, Clayton Yang Hashim Ahmed, Kelly Avery, Jane Blazeby, Natalie Blencowe, Richard Bryant, David Chang, Sian Cousins, Michael Douek, Christin Hoffman, David Jayne, Connor Jones, Rhiannon Macefield, Barry Main, Samir Pathak, Shelley Potter, Arnie Purushotham, Grant Stewart, Danail Stoyanov, Jayant Vaidya, Tom Vercauteren, Dale Vimalachandran, Oliver Brewster, Manuk Wijeyaratne

**Affiliations:** Centre for Surgical Research, Population Health Sciences, University of Bristol, 39 Whatley Road, Clifton, Bristol, BS8 2PS UK

**Keywords:** Fluorescence imaging, Indocyanine green, Surgical oncology

## Abstract

**Background:**

Fluorescence-guided precision cancer surgery may improve survival and minimize patient morbidity. Efficient development of promising interventions is however hindered by a lack of common methodology. This methodology review aimed to synthesize descriptions of technique, governance processes, surgical learning and outcome reporting in studies of fluorescence-guided cancer surgery to provide guidance for the harmonized design of future studies.

**Methods:**

A systematic search of MEDLINE, EMBASE and CENTRAL databases from 2016–2020 identified studies of all designs describing the use of fluorescence in cancer surgery. Dual screening and data extraction was conducted by two independent teams.

**Results:**

Of 13,108 screened articles, 426 full text articles were included. The number of publications per year increased from 66 in 2016 to 115 in 2020. Indocyanine green was the most commonly used fluorescence agent (391, 91.8%). The most common reported purpose of fluorescence guided surgery was for lymph node mapping (195, 5%) and non-specific tumour visualization (94, 2%). Reporting about surgical learning and governance processes incomplete. A total of 2,577 verbatim outcomes were identified, with the commonly reported outcome lymph node detection (796, 30%). Measures of recurrence (32, 1.2%), change in operative plan (23, 0.9%), health economics (2, 0.1%), learning curve (2, 0.1%) and quality of life (2, 0.1%) were rarely reported.

**Conclusion:**

There was evidence of methodological heterogeneity that may hinder efficient evaluation of fluorescence surgery. Harmonization of the design of future studies may streamline innovation.

**Supplementary Information:**

The online version contains supplementary material available at 10.1186/s12885-024-12386-4.

## Introduction

Improving surgical precision is a key challenge in cancer surgery. The ability to precisely map boundaries between cancerous and normal tissues intra-operatively is important to optimize complete R0 resection rates to minimize local disease recurrence [1], improve survival [2], avoid re-intervention or the need for adjuvant therapy [3], and reduce costs [4]. Greater precision may also reduce damage to adjacent health tissues thereby minimizing morbidity and functional loss, and lead to improved quality of life. Precision surgery is therefore considered a major research priority for patients, researchers and funding organizations [5, 6].

Near-infrared (NIR) fluorescence is an emerging technique which may have a wide range of clinical benefits for intra-operative tumour visualization, oncological margin control, lymph node mapping, as well as vital structure delineation, and assessment of tissue vascularity or viability [7]. There has been continual development of fluorescent agents, imaging systems, and their applications over the past several decades [8]. There remains however tremendous variability in the administration of these agents, as well as numerous other questions regarding technical and governance aspects of their use [9]. In such rapidly expanding fields there is a need for accelerated research to aid clinical translation.

Clinical research can be accelerated to tackle pressing research priorities [10] and this may be achieved through the use of “master” [11, 12] or “core protocols” [10] to act as a blueprint to investigate multiple hypotheses through concurrent sub-studies. This approach has a modular structure to account for different diseases or interventions, with central generic components to streamline delivery [13, 14]. Typically, core protocols are defined as including novel randomized trial designs such as basket, umbrella, or platform trials and are most common in phase II/III drug trials [13, 15, 16], however, the benefits of a core protocol may extend to other settings. For example, core protocols have been applied widely in studies of precision oncology [17, 18] but are rare when investigating surgical therapies and no core protocol exists for precision cancer surgery. Surgical interventions are developed differently to medicines [19] and pre-trial surgical research may benefit from creating a ‘core translational protocol’ to streamline development of surgical innovation and seamlessly segue into randomized evaluation. Such a protocol may include, for example, standardized development cycles, outcome measures, quality assurance processes, and participant-level data sharing agreements.

The CLEARER (Cancer fLuorescencE imAge-guided suRgERy) Collaboration brings together diverse multi-disciplinary professional and patient stakeholders to inform the development of a core translational protocol for NIR fluorescence-guided precision cancer surgery. This review aims to critically synthesize methodology and outcome selection in studies of NIR fluorescence guided cancer surgery to provide guidance and recommendations for the harmonized design of future studies. Specifically, it will synthesize: 1) descriptions of NIR surgical interventions, 2) surgical learning and governance processes and 3) outcome selection and measurement across all diseases and procedures that use NIR techniques to inform the development of a core outcome set.

## Methods

The systematic review protocol is registered in the International Prospective Register of Systematic Reviews (http://www.crd.york.ac.uk/PROSPERO) (CRD42021243401) [12]. The review was performed in accordance with the preferred reporting items for systematic review and meta-analyses guidelines [20].

### Eligibility criteria

All observational (case report, case series, cross-sectional, case–control, cohort) and interventional (randomised controlled, non-randomised controlled, community trials) studies in which human participants with malignant neoplasms undergo surgery for the treatment of primary or secondary malignancy with the intra-operative use of NIR-fluorescence imaging were eligible for inclusion. Editorials, news, comments, conference proceedings, video papers, study protocols and letters were excluded, as were studies in non-human participates or those in haematological malignancies (as these are not surgically managed) or non-melanoma skin cancer (as commonly excluded from national cancer registries and cancer databases). Studies investigating in vitro-surgery or those reporting outcomes of delayed reconstruction following cancer surgery were also excluded.

### Search strategy

A systematic electronic search was performed of MEDLINE (via OvidSP), EMBASE (via OvidSP), and the Cochrane Central Register of Controlled Trials (CENTRAL) from 1st January 2016 to 31st December 2020. A search strategy combined appropriate search terms for “surgery”, “cancer” and “near-infrared fluorescence “ adapted for each database. Searches were externally peer reviewed according to the PRESS Guideline Statement and are referenced in Supplementary Materials [21]. The search output was de-duplicated according to established methods and uploaded to a web-based screening tool.

### Study selection

Titles and abstracts were screened by two independent researchers for eligibility with discrepancies resolved by a third researcher. All potentially eligible full text articles were further assessed and reasons for exclusion documented.

### Data extraction

Full text data extraction for each article was completed on an electronic database (REDCap) using standard proforma accompanied by guidance notes. All articles were independently double reviewed for quality assurance purposes with discrepancies resolved by a third reviewer. Basic citation details including lead author name, publication year and journal, funding arrangements and conflicts of interest were extracted. Study design was determined using methods described by Grimes et al*.* [22]. Descriptions of surgical procedures studied as co-interventions with NIR technology were extracted verbatim (e.g. “right hemicolectomy”). Verbatim text was reviewed by two surgeons and independently grouped into overarching categories (e.g. “colonic resections”) and summarised by clinical specialty (e.g. colorectal surgery). Discrepancies between reviewers were resolved through discussion with the study team.

Data was extracted across three themes, namely, 1) descriptions of NIR surgical interventions, 2) surgical learning and governance processes and 3) outcome selection and measurement. Details are summarised below with the full data extraction form presented in Supplementary Materials.

1. Descriptions of NIR surgical interventions

Details of study aims, interventions, comparators (where applicable), clinical and demographic participant data were recorded. This incorporated specific data about NIR surgery including the type of fluorescence agent used, the manufacturer, dose and technique of constitution, number of time points that the fluorescence agent was administered and assessed, and details regarding the model of imaging system, type of display and quantitative analysis. The purpose of NIR guided surgery was classified as: lymph node mapping (for example, sentinel node identification or assessment of completeness of lymph node dissection), specific tumour visualisation (highlighting tumours by binding to specific markers on tumour surface e.g. antigens or integrins), non-specific tumour visualization (highlighting tumour without binding to specific markers), vascularisation around tumour (for example, to reduce damage to surrounding vascular structures), vascular supply to the tumour (for example to guide vessel clamping) and vascularisation for tissue reconstruction. Author’s descriptions of surgical procedures were extracted verbatim and grouped in speciality.

2. Surgical learning and governance processes

Details describing any reported surgical learning, or methods to address a reported learning curve were similarly documented. Extracted governance processes included documented ethics committee approval, clinical trials registration, and consent processes. If reported, the number of patients declining the intervention was recorded as a measure of patient acceptability.

3. Outcome selection and measurement

Study outcomes, outcome definitions, method of measurement, assessor, time, and unit of measurement were extracted verbatim through line-by-line coding of textual data including tables and appendices. Outcomes were categorised into domains using an inductive approach. At least two independent researchers read and re-read extracted outcomes for familiarisation and categorised outcomes thematically. Domains were generated and refined an iterative process that compared initial themes with new themes that emerged as the analysis progressed. Dual domain categorisation was reconciled by a third independent team of authors. Outcome reported was further assessed according to the COHESIVE core outcome set framework [23]. This core outcome set defines 8 domains to be measured in early phase studies of surgical innovation and includes measures of the intended benefits (e.g. less operative time), modifications to the procedure, procedure completion success (e.g. the technical steps were completed as planned), problems with device working (where applicable), expected and unexpected disadvantages, the overall desired effect of the procedure (e.g. tumour successfully excised), operators’ experiences (e.g. ergonomic comfort), and patients’ experiences.

### Data

Descriptive statistics were used to summarise extracted data and demonstrate areas of heterogeneity that may benefit from harmonization. Data were organised by year to examine the evolution of NIR guided cancer surgery over time. A narrative summary was created to describe potential areas of harmonisation. No meta-analysis was performed as this was a methodology review and did not aim to estimate treatment effects.

## Results

**Fig. 1:**
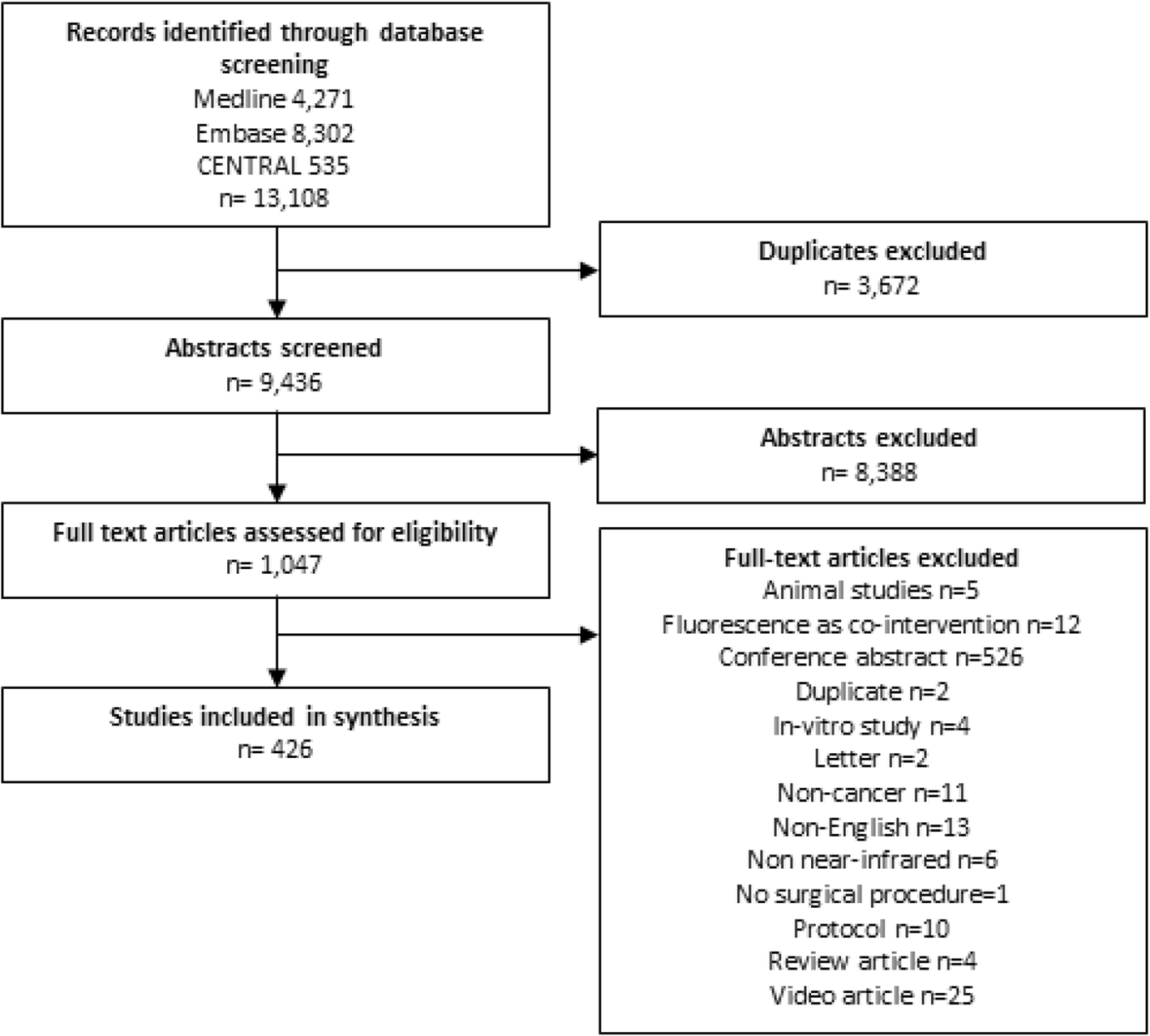
Prisma flow chart

There were 13,108 records identified through database screening. Of these records, 426 full text articles were included in the final analysis (Fig. 1).

### Study characteristics

Details of included studies are presented in Table 1. Numbers of publications per year increased from 66 in 2016 to 115 in 2020. Most studies had an Asia pacific (173, 40.6%) or European (134, 31.5%) setting, and investigated the use of NIR guided surgery in patients with gynaecological (103, 24.3%), lower gastrointestinal (75, 17.6%), hepato-pancreato-biliary (49, 11.5%), and oesophagogastric surgery (44, 10.3%). There were few randomised trials (19, 4.5%), and most were single centre (246, 57.8%), descriptive (316, 74.2%) studies, without comparators, with a median sample size of 31 (range 1–1079). The most common comparator was surgery without fluorescence (61/110, 57.3%).

**Table 1:** Study characteristics (*N* = 426)

	**N**	**%**
**Year of publication**		
2016	66	15.5
2017	64	15
2018	87	20.4
2019	94	22.1
2020	115	27
**Geographical region**		
Asia Pacific	173	40.6
North America	71	16.7
Europe	134	31.5
South Asia/Middle East	10	2.4
Multiple	38	8.9
**Specialty of study**		
Breast	31	7.3
Gynaecology	103	24.2
Head and Neck	34	8
Hepato-Pancreatico-Biliary	49	11.5
Lower GI	75	17.6
Neurology	14	3.3
Paediatrics	5	1.2
Lung	34	8
Oesophagogastric	44	10.3
Urology	24	5.6
Other	5	1.2
Multiple	8	1.9
**Centres**		
Single centre	246	57.8
Multi centre	42	9.9
Not reported	138	32.4
**Type of study design**		
Randomised trial	19	4.5
Non-randomised trial	18	4.2
Case cohort study	68	16
Case control study	5	1.2
Descriptive	316	74.2
**Comparisons**		
Comparative study	110	25.8
Compared with no fluorescence	63	57.3
Comparative NIR agent	5	4.6
Comparative NIR technique	8	7.3
Comparative NIR dose	13	11.8
Comparative co-intervention	6	5.5
Comparative patient group	11.8	0.9
Other	14	12.7
**Sample size; median (range)**	31	(1–1079)

### Descriptions of NIR surgical interventions

**Fig. 2:**
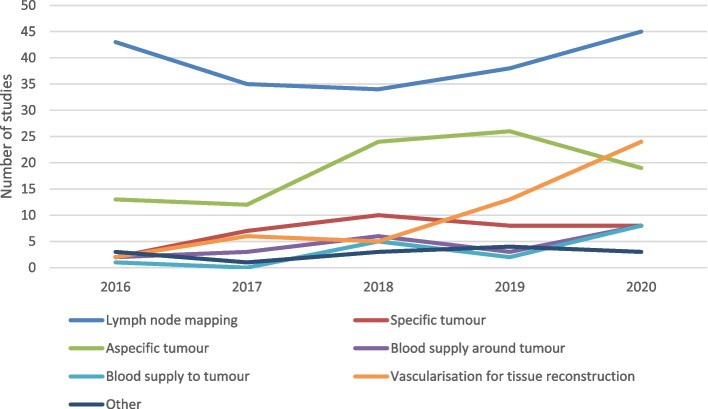
Reported purpose of NIR guided cancer surgery in included studies by year

The most common reported purpose of NIR guided surgery was for lymph node mapping (195 studies, 46%) and non-specific tumour visualization (94 studies, 22%, Fig. 2). Studies investigating NIR guided surgery for tissue reconstruction increased from 2 studies in 2016 to 24 studies in 2020, largely driven by studies of gastrointestinal cancer surgery.

The most common reported surgical procedures are presented in Table 2. Procedures were categorized into 86 groups including studies that reported specific procedures (e.g. NIR guided gastrectomy; 26 studies) or a heterogeneous mixture of procedures (e.g. colon and rectal resections (various), 26 studies). Sentinel lymph node biopsy and lymph node dissection were common across multiple cancer types.

**Table 2:** Most common reported surgical procedures by cancer type (*N* = 426)

Breast cancer n (%)	31	
Sentinel lymph node biopsy	17	(55)
Breast cancer resections (various)	7	(23)
Wide local excision	2	(6)
Other	5	(16)
Hepato-pacreatico-biliary cancer n (%)	49	
Liver resection	27	(55)
Pancreatic resection	13	(27)
Staging laparoscopy	2	(4)
Other	7	(14)
Lower gastrointestinal cancer n (%)	75	
Colonic and rectal resection (various)	26	(35)
Anterior resection of the rectum	17	(23)
Colonic resection	7	(9)
Other	25	(33)
Neurological cancer n (%)	14	
Primary brain tumour resection	10	(71)
Brain metastases resection	1	(7)
Other	3	(21)
Lung cancer n (%)	34	
Segmentectomy	12	(35)
Lung resection (various)	9	(26)
Wedge resection	5	(15)
Other	8	(24)
Oesophagogastric cancer n (%)	44	
Gastrectomy	26	(59)
Oesophagectomy	13	(30)
Other	5	(11)
Urological cancer n (%)	24	
Partial nephrectomy	7	(29)
Sentinel lymph node biopsy	4	(17)
Prostatectomy	3	(13)
Other	10	(41)
Gynaecological cancer n (%)	104	
Hysterectomy ± bilateral salpingoopherectomy ± lymph node dissection	75	(74)
Other	29	(26)
Head and neck cancer n (%)	34	
Head/neck cancer resection (various)	14	(41)
Neck dissection	4	(12)
Sentinel lymph node biopsy	4	(12)
Other	12	(35)
Paediatric cancer n (%)	5	
Lung resection	1	(20)
Cancer resection (various)	3	(60)
Peritoneal metastases resection	1	(20)
Other n (%)	5	
Adrenalectomy	2	(40)
Sentinel lymph node biopsy for melanoma	1	(20)
Sarcoma resection	2	(40)
Multiple cancers n (%)	8	
Mixed procedures	2	(25)
Mixed site sentinel lymph node biopsy	2	(25)
Cytoreductive surgery	1	(13)
Other	3	(38)

Details about NIR administration, data capture and analysis are presented in Table 3. Most studies reported using NIR in real time (343 studies, 80.5%), with indocyanine green (ICG) the most commonly used fluorescence agent (391, 91.8%), used without labelling (389, 86%). Overall, reporting fluorescence dose (387 studies, 90.1%) and system model (341, 80.1%) was widespread. Agents were usually administered once (372, 87.3%) and most often directly into tissues (220, 51.6%). Studies most frequently assessed fluorescence either once (166, 29%) or four times (170, 39.9%), typically without a control (297, 69.7%). Most studies did not report details on methods to display fluorescence (367, 86.6%).

**Table 3:** Reported details of NIR administration, data capture and analysis (*n* = 426)

N		%
**Real-time use of fluorescence imaging**	343	80.5
**Type of fluorescent agent used**		
ICG	391	91.8
MB	3	0.7
IRDye800CW	14	3.3
OTL38	8	1.9
SGM-101	4	0.9
ZEOCLIP	3	0.7
Other	3	0.7
**Dose of fluorescence agent**	387	90.9
**Fluorescence manufacturer**	213	50.0
**Number of timepoints fluorescence was administered**		
1	372	87.3
2	42	9.9
3 +	11	2.6
Not stated	1	0.2
**Mode of administration**		
Intravenous	197	46.2
Into tissue	220	51.6
Not stated	9	2.1
**Number of times fluorescence was assessed**		
1	166	29.0
2	58	13.6
3	28	6.6
4	170	39.9
5 +	4	0.9
**Manufacturer of NIR system**		
Karl Storz	85	24.9
Stryker	62	18.2
Intuitive	39	11.4
Hamamatsu	29	8.5
Medtronic	20	5.9
Quest	12	3.5
Olympus	10	2.9
Fluoptics	9	2.6
Mizuho	7	2.1
Other	39	11.5
Mixed	28	8.2
Not stated	85	19.9
**How fluorescence was assessed intra-operatively**		
Visual appearance (no control)	297	69.7
Visual appearance (control)	31	7.3
Quantified	26	6.1
Other	72	16.9
**Use of fluorescence imagining overlayed on white light display**		
Yes	76	17.8
No	30	7.0
Not stated	320	75.1
**Intraoperative display**		
Standard screen	29	6.8
3D screen	4	0.9
Immersive (e.g. da Vinci console)	20	4.7
Other	4	0.9
Not stated	367	86.6
**Labelling of fluorescence with**		
Antibody	15	3.5
Nanoparticle	2	0.5
Nanocolloid	16	3.8
Radiolabelled	12	2.8
Other	12	2.8
None	369	86.6

*Abbreviations/explanations*: *ICG* indocyanine green, *MB* methylene blue, *IRDye800CW* near-infrared fluorophore, *OTL38* folate-indole-cyanine green-like conjugate to folate receptor alpha (FRa), *SGM* antibody-dye conjugate to carcinoembryonic antigen (CEA) with a 700 nm fluorochrome, *ZEOCLIP* endoscopic fluorescent clip

### Surgical learning and governance processes

The number of studies reporting fluorescence-related or generic details about indicators of surgical learning and any governance processes are presented in Table 4. Surgical learning was reported in 40 out of 425 (9.4%) included studies. Most common was reporting generic statements about surgeon experience (94, 22.1%) but few described experience of NIR guided surgery, training received (10, 2.4%), or the use of proctorship (4, 0.9%). Information about usual caseloads (including relevant to hospital, surgeon, fluorescence uses or in general) were provided in less than 4% of studies, respectively.

**Table 4:** Reporting of details related to surgical learning and governance processes (*N* = 426)

	**N**	**%**
**Surgical learning**		
Assessed or described surgical learning	40	9.4
Reported training surgeons received prior to first in-human procedure	10	2.4
Defined criteria for surgeon eligibility	22	5.2
Reported number of surgeons	90	21.1
Reported use of proctorship	4	0.9
Reported usual caseload for surgeon		
with fluorescence	6	1.4
without fluorescence	7	1.64
Reported usual caseload for centre		
with fluorescence	13	3.1
without fluorescence	14	3.3
Generic statement of surgical experience	94	22.1
Grade of surgeon	25	5.9
Usual caseload for surgeon without fluorescence of procedure of interest	7	1.64
**Governance processes**		
Studies reporting consecutive patients	112	26.3
Reported inclusion and exclusion criteria	256	60.1
Details of patients not meeting inclusion criteria	13	3.1
Number of patients declining intervention	18	4.2
Reported regulator approval (FDA/NICE/CE marking/other clinical effectiveness regulator)	47	11.0
Reported individual patient consent	318	74.7
Conflict of interest statement		
Declared no conflict of interest	316	74.2
Declared conflict of interest	63	14.8
No conflict of interest statement reported	47	11
Funding statement		
Funding received	163	38.3
No funding received	99	23.2
No funding statement reported	164	38.7
Statement confirming IRB*/ethics committee approval	353	82.9
Amendment to the IRB/ethics approval AFTER the study had started	2	0.5
Independent committee oversight	4	0.94
Prior registration with trials register	89	20.9

*Abbreviations*: *FDA* United States Food and Drug Administration /*NICE* The National Institute for Health and Care Excellence /*CE* Conformity with European health, safety, and environmental protection standards, *IRB* institutional review board

Reporting about governance processes was incomplete. A total of 318 (75%) documented individual patient consent, 352 (83%) confirmed research ethics committee approval and 256 (60%) reported inclusion and exclusion criteria.

### Outcome selection and measurement

A total of 2,577 verbatim outcomes were identified from included articles and were categorised into eight outcome domains and 39 subdomains (Table 5). The most commonly reported outcome was lymph node detection (796 verbatim outcomes, 30%), followed by non-fluorescence related adverse events (277, 10.7%), histological assessment of tissue (192, 7.5%) and tumour detection (173, 6.7%). Measures of recurrence (32, 1.2%), survival (53, 2.1%), change in operative plan (23, 0.9%), health economics (2, 0.1%), learning curve (2, 0.1%) and quality of life (2, 0.1%) were rarely reported.

**Table 5:** Frequency of outcome reporting by outcome domains and sub-domains (*N* = 2552)

Outcome domain	Outcome sub-domains	n	%
**NIR-specific outcomes**	Lymph node detection	796	30.9
	Tumour detection (intra-operative)	173	6.7
	ICG administration outcomes	126	4.9
	Fluorescence specific adverse events	60	2.3
	Resection margins (histopathological)	53	2.1
	Fluorescence intensity (tumour to background ratio)	43	1.7
	Fluorescence visualisation	30	1.2
	Vascularity assessment	26	1
	Non-specific feasibility/accuracy of NIR	24	0.9
	Change in operative plan	23	0.9
	Structure identification	17	0.7
	Depth of fluorescence visualisation	9	0.3
	Pharmacokinetics	5	0.2
	Interobserver agreement in fluorescence	2	0.1
**Adverse events**	Adverse event—not fluorescence related	277	10.7
	Anastomotic leak	43	1.7
	Blood transfusion	13	0.5
	Non-specific safety outcome	12	0.5
	Re-operation	12	0.5
	Re-admission	5	0.2
**Intra-operative outcomes**	Length/timing of procedure	135	5.2
	Blood loss	84	3.3
	Intra-operative event/descriptor	82	3.2
	Non-specific descriptions of success	10	0.4
	Vascularity assessment (not NIR guided)	6	0.2
**Oncology outcomes**	TNM stage	192	7.5
	Recurrence	32	1.2
	Survival	53	2.1
**Health economic**	Cost	2	0.1
**Patients' experience**	Patients' experience	3	0.1
	Quality of life	2	0.1
**Post operative outcomes**	Length of stay	83	3.2
	Descriptions of the post operative course	56	2.2
	Post-operative physiological measure	44	1.7
**Surgeon**	Learning curve	2	0.1
	Surgeons' perception of the procedure	4	0.2

Most studies (405, 95%) reported a measure that the NIR guided surgery was completed successfully (Table 6). Less than a quarter of studies described modifications to the procedure, unexpected disadvantages, surgeons’ or patients’ experiences of the procedure.

**Table 6:** Reporting of COHESIVE core outcome set domains (*N* = 426)

**Reported measurement domain**	N	%
Successful procedure completion	402	94.4
Overall desired effect achieved	365	85.7
Modifications to the procedure	45	10.6
Problems with the device working	16	3.8
Intended benefits of the procedure	365	85.7
Expected disadvantages of the procedure	95	22.3
Unexpected disadvantages of the procedure	57	13.4
Surgeons’ experiences of the procedure	29	6.8

## Discussion

This review provides a comprehensive methodological summary of studies of NIR-guided surgery for cancer. It demonstrates that NIR-guided surgery is an expanding field with uses across the spectrum of solid organ tumours and has been used to augment a wide range of established procedures. While most studies used non-cancer specific ICG fluorescence, other agents and specific labelling, such as those to carcinoembryonic antigen [24–26], are now being used. This is consistent with other reviews in the field [27]. The potential benefits of NIR-guided surgery are relevant across cancer types and include improved accuracy of lymph node harvest and tumour margin detection. There was, however, evidence of heterogenous reporting of NIR interventions, surgical learning, governance processes and outcomes that hinders efficient evaluation of NIR surgery. This suggests that harmonisation of methodology may be appropriate.

Guidelines exist to describe the development of surgical innovation and implementation into clinical practice [28, 29]. These refer to cycles through which innovations are iteratively tested, modified and refined. Efficient development processes are dependent on appropriate descriptions of the intervention so they can be replicated and improved. This review demonstrates that innovation descriptions in NIR-guided surgery for cancer are both heterogenous and deficient. Standardized methods and reporting for procedural aspects such as quantification [30, 31], margin assessment [32] and dosing [33] have been recently suggested and could be used in future studies to improve methodological homogeneity.

Appropriate oversight and governance is required for innovation to occur transparently and safely [34]. Governance reporting in both this review and other systematic reviews of the introduction of an innovative procedure have demonstrated inadequate reporting in multiple domains including funding information, patient consent and reporting of the number of patients declining the intervention [35]. The procedural learning curve, although rarely discussed in the included studies, is an important consideration in an innovative surgical technique. Any new procedure which is a variant from the current standard of care may require a period of training to achieve satisfactory performance [36]. The learning curve related to fluorescent guided oncological surgery is not currently known.

The majority of the outcomes presented in included studies were short-term clinical and technical outcomes. This is similar to other recent systematic reviews of outcome reporting in innovative surgical procedures [35, 37, 38]. Although this establishes the sensitivity and specificity of the imaging agent and reports immediate adverse reactions, it does not demonstrate its potential to improve oncological and functional outcomes in clinical practice. Providing insight into how a new technique affects patient care is essential for funding agencies and regulatory bodies. The lack of reporting of the clinical impact of these techniques may hamper their widespread implementation by facilitating the move from early phase studies to randomised controlled trials. Reporting of multiple aspects of the innovation-specific COHESIVE outcomes domains were also poorly reported. These recently established guidelines include factors critical to the implementation of new devices and technologies into clinical practice. Previous studies have assessed outcome reporting in magnetic augmentation of the lower oesophageal sphincter [38] and minimally invasive liver resection [35] and similarly demonstrated poor adherence to reporting guidelines, in this case the IDEAL framework [29]. Lack of adequate innovation-specific outcome reporting for new surgical technologies may result in individual surgeons or units repeating ineffective or even harmful modifications [39].

Since its inception the use of fluorescence imaging, with ICG in particular, has expanded exponentially [9]. This review however highlights a lack of research progression as the majority of studies over a five year period were single-centre, descriptive case series with a small number of included patients. A recent survey demonstrated lack of confidence in the current evidence to be the primary barrier to more widespread adoption [40]. It therefore may be time to change the way in which we conduct research in fluorescence-guided cancer surgery with a move toward novel study designs. Master protocols increase the efficiency of clinical research and reduce duplication and waste [11]. Master protocols are well recognised in oncology [41], yet are less well established in other research fields, and there are currently no master protocols to investigate surgical techniques. Unlike a traditional clinical trial, which investigates a single treatment for a group of relatively homogenous patients, a platform trial is designed to simultaneously investigate multiple treatments for a disease or a group of closely related diseases [13]. A platform trial using a core master protocol which unites a common aspect of fluorescence guided surgery, such as the intra-operative identification of lymph nodes, could be applied across various cancer sites [11]. Existing reporting guidelines for fluorescence surgery [31, 42, 43] as well as innovation specific reporting guidelines could be incorporated [23, 29]. This would potentially establish a large trial network with a common infrastructure across and within multiple institutions, and allow the incorporation of new technologies as they emerge. Using a collective methodology and data reporting system would generate high-quality research outputs which answer multiple questions concurrently [10].

Rigorous methods were used to identify relevant studies, and categorise data and outcomes in this cross-speciality methodological study in near-infrared fluorescence cancer surgery. This review however has several limitations. The study period was restricted to five years up to 2020. This may have resulted in a disproportionate number of studies in a particular speciality, such as gynaecology, as research in fluorescence surgery in this speciality hit a peak of popularity. Assessing the effect size of fluorescent guided cancer surgery was out with the scope of this review. For this reason, a risk of bias assessment was not performed for included studies. It however may have been useful to compare methodological aspects of studies with varying degrees of bias. Similarly, the search was not updated prior to publication because the aim of the review was to synthesise methodology not estimate effect size. A sample of at least 100 studies is generally recommended [44], beyond which further data collection is unlikely to yield meaningful insights. It is acknowledged that an update may, however, show some differences. To establish a master protocol in fluorescence guided surgery further methodological research is required. A platform to allow shared learning will allow surgeons to describe the innovation in real time along with any modifications. Infrastructure to streamline governance to allow individual patient-level data sharing would facilitate this. Key stakeholders should be involved to agree upon key quality assurance processes as well as outcome measures and reporting standards. This may involve the construction of a core outcome set specific to NIR-infrared fluorescence guided surgery.

Finally, the incorporation and acceptance into clinical practice of fluorescence guided surgery is hampered by inadequate reporting of the surgical intervention, surgical learning, and governance processes, and heterogeneity in outcome selection and measurement. A master protocol may harmonise methodology and reporting across this rapidly evolving technology.

### Supplementary Information

Supplementary Material 1.

Supplementary Material 2.

## Data Availability

The datasets generated and analyzed during the current study are available from the corresponding author upon reasonable request.

## References

[1] Ghaneh P (2019). The impact of positive resection margins on survival and recurrence following resection and adjuvant chemotherapy for pancreatic ductal adenocarcinoma. Ann Surg.

[2] Meric F (2003). Positive surgical margins and ipsilateral breast tumor recurrence predict disease-specific survival after breast-conserving therapy. Cancer Interdiscipl Int J Am Cancer Soc.

[3] Grossfeld GD (2000). Impact of positive surgical margins on prostate cancer recurrence and the use of secondary cancer treatment: data from the CaPSURE database. J Urol.

[4] Orosco RK (2018). Positive surgical margins in the 10 most common solid cancers. Sci Rep.

[5] Commission on the Future of Surgery — Royal College of Surgeons. https://futureofsurgery.rcseng.ac.uk/report/Future%20of%20Surgery%20Report.pdf. Accessed May 2023.

[6] Terry SF. Obama's Precision Medicine Initiative. Genet Test Mol Biomarkers. 2015;19(3):113-4. 10.1089/gtmb.2015.1563. 10.1089/gtmb.2015.1563 PMC4361161 25751403

[7] Stammes MA (2018). Modalities for image- and molecular-guided cancer surgery. Br J Surg.

[8] Van Keulen S, Hom M, White H, Rosenthal EL, Baik FM. The Evolution of Fluorescence-Guided Surgery. Mol Imaging Biol. 2023;25(1):36-45. 10.1007/s11307-022-01772-8. 10.1007/s11307-022-01772-8 PMC9971137 36123445

[9] Dip F (2022). Consensus conference statement on the general use of near-infrared fluorescence imaging and Indocyanine green guided surgery: results of a modified Delphi Study. Ann Surg.

[10] Park JJH (2021). How COVID-19 has fundamentally changed clinical research in global health. Lancet Glob Health.

[11] Woodcock J, LaVange LM (2017). Master protocols to study multiple therapies, multiple diseases, or both. N Engl J Med.

[12] Park JJH (2021). Randomised trials at the level of the individual. Lancet Glob Health.

[13] Park JJH (2019). Systematic review of basket trials, umbrella trials, and platform trials: a landscape analysis of master protocols. Trials.

[14] Coyle C (2016). ADD-ASPIRIN: A phase III, double-blind, placebo controlled, randomised trial assessing the effects of aspirin on disease recurrence and survival after primary therapy in common non-metastatic solid tumours. Contemp Clin Trials.

[15] Meyer EL (2020). The evolution of master protocol clinical trial designs: a systematic literature review. Clin Ther.

[16] Siden EG (2019). Reporting of master protocols towards a standardized approach: a systematic review. Contemp Clin Trials Commun.

[17] Park JJH (2020). An overview of precision oncology basket and umbrella trials for clinicians. CA Cancer J Clin.

[18] Chan KKW (2021). The past, present, and future of economic evaluations of precision medicine at the committee for economic analyses of the Canadian cancer trials group. Curr Oncol.

[19] Hirst A, Philippou Y, Blazeby J, Campbell B, Campbell M, Feinberg J, Rovers M, Blencowe N, Pennell C, Quinn T, Rogers W, Cook J, Kolias AG, Agha R, Dahm P, Sedrakyan A, McCulloch P. No Surgical Innovation Without Evaluation: Evolution and Further Development of the IDEAL Framework and Recommendations. Ann Surg. 2019;269(2):211-20. 10.1097/SLA.0000000000002794. 10.1097/SLA.0000000000002794 29697448

[20] Shamseer L (2015). Preferred reporting items for systematic review and meta-analysis protocols (PRISMA-P) 2015: elaboration and explanation. BMJ : Bri Med J.

[21] McGowan J (2016). PRESS Peer Review of Electronic Search Strategies: 2015 guideline statement. J Clin Epidemiol.

[22] Grimes DA, Schulz KF (2002). An overview of clinical research: the lay of the land. Lancet.

[23] Avery KNL, Wilson N, Macefield R, McNair A, Hoffmann C, Blazeby JM, Potter S. Core Outcomes for early pHasE Surgical Innovation and deVicEs (COHESIVE) study steering group. A Core Outcome Set for Seamless, Standardized Evaluation of Innovative Surgical Procedures and Devices (COHESIVE): A Patient and Professional Stakeholder Consensus Study. Ann Surg. 2023;277(2):238-45. 10.1097/SLA.0000000000004975. 10.1097/SLA.0000000000004975 PMC9831031 34102667

[24] de Valk KS, Deken MM, Schaap DP, Meijer RP, Boogerd LS, Hoogstins CE, van der Valk MJ, Kamerling IM, Bhairosingh SS, Framery B, Hilling DE, Peeters KC, Holman FA, Kusters M, Rutten HJ, Cailler F, Burggraaf J, Vahrmeijer AL. Dose-Finding Study of a CEA-Targeting Agent, SGM-101, for Intraoperative Fluorescence Imaging of Colorectal Cancer. Ann Surg Oncol. 2021;28(3):1832-44. 10.1245/s10434-020-09069-2. 10.1245/s10434-020-09069-2 PMC7892528 33034788

[25] Schaap DP (2020). Carcinoembryonic antigen-specific, fluorescent image-guided cytoreductive surgery with hyperthermic intraperitoneal chemotherapy for metastatic colorectal cancer. Br J Surg.

[26] Hoogstins CES (2018). Image-guided surgery in patients with pancreatic cancer: first results of a clinical trial using SGM-101, a novel carcinoembryonic antigen-targeting, near-infrared fluorescent agent. Ann Surg Oncol.

[27] Sutton PA (2023). Fluorescence-guided surgery: comprehensive review. BJS Open.

[28] Skivington K (2021). A new framework for developing and evaluating complex interventions: update of medical research council guidance. BMJ.

[29] McCulloch P (2009). No surgical innovation without evaluation: the IDEAL recommendations. Lancet.

[30] Hoogstins C (2019). Setting standards for reporting and quantification in fluorescence-guided surgery. Mol Imag Biol.

[31] Tummers WS (2018). Recommendations for reporting on emerging optical imaging agents to promote clinical approval. Theranostics.

[32] Steinkamp PJ (2021). A standardized framework for fluorescence-guided margin assessment for head and neck cancer using a tumor acidosis sensitive optical imaging agent. Mol Imaging Biol.

[33] Wakabayashi T (2022). Indocyanine green fluorescence navigation in liver surgery: a systematic review on dose and timing of administration. Ann Surg.

[34] Blazeby JM, Cousins S, Pullyblank A (2022). Safety and transparency in surgical innovation. Br J Hosp Med.

[35] Pathak S (2021). A systematic review of minimally invasive Trans-thoracic liver resection to examine intervention description, governance, and outcome reporting of an innovative technique. Ann Surg.

[36] Papachristofi O, Jenkins D, Sharples LD (2016). Assessment of learning curves in complex surgical interventions: a consecutive case-series study. Trials.

[37] Wilson N (2022). Identification of outcomes to inform the development of a core outcome set for surgical innovation: a targeted review of case studies of novel surgical devices. BMJ Open.

[38] Kirkham E (2020). Systematic review of the introduction and evaluation of magnetic augmentation of the lower oesophageal sphincter for gastro-oesophageal reflux disease. J Bri Surg.

[39] Angelos P (2013). Ethics and surgical innovation: challenges to the professionalism of surgeons. Int J Surg.

[40] Verhoeff K, Mocanu V, Fang B, Dang J, Sun W, Switzer NJ, Birch DW, Karmali S. Characterization of Near-Infrared Imaging and Indocyanine-Green Use Amongst General Surgeons: A Survey of 263 General Surgeons. Surg Innov. 2022;29(4):494-502. 10.1177/15533506221094962. 10.1177/15533506221094962 PMC9527369 35451339

[41] Hirakawa A (2018). Master protocol trials in oncology: Review and new trial designs. Contemp Clin Trials Commun.

[42] Lauwerends LJ (2021). Real-time fluorescence imaging in intraoperative decision making for cancer surgery. Lancet Oncol.

[43] Pogue B (2018). Perspective review of what is needed for molecular-specific fluorescence-guided surgery. J Biomed Opt.

[44] Whistance RN (2013). A systematic review of outcome reporting in colorectal cancer surgery. Colorectal Dis.

