# Supplementary material for: A systematic methodology review of fluorescence-guided cancer surgery to inform the development of a core master protocol and outcome set
Source: BMC Cancer. 2024 Jun 7;24:697. doi: 10.1186/s12885-024-12386-4 (PMC11157717; doi:10.1186/s12885-024-12386-4)
Supplement: Supplementary file 2 — Supplementary Material 2. [file 12885_2024_12386_MOESM2_ESM.docx]

**Appendix 2- Data extraction form**

| Q | Branching | Question | Options |
| --- | --- | --- | --- |
| **Form A: Citation details and funding arrangements.** | | | |
| 1 |  | 1^st^ Author |  |
| 2 |  | Publication year |  |
| 3 |  | PubMed reference.  *Please include full reference if possible e.g. Su, H., et al., Indocyanine green fluorescence imaging to assess bowel perfusion during totally laparoscopic surgery for colon cancer. BMC Surgery, 2020. 20(1): p. 102.* |  |
| 4 |  | Assessor name |  |
| 5 |  | Date of entry |  |
| 6a |  | Does the article provide a conflict of interest statement (Including “no conflict of interest”) | Yes  No |
| 6b | If yes, 6a | Do any of the authors have conflicts of interest? | Yes  No |
| 6c | If yes, 6a | Record any statement about conflict of interest (including declarations of no COI) including which author(s) it refers to and the precise nature of the COI here: |  |
| 7a |  | Did the study receive funding and/or sponsorship? | Yes  No  Not reported |
| 7b | If yes, 7a | What was the source of funding or sponsorship? (Copy verbatim) |  |
| **Section B: Study design and rationale** | | | |
| 1 |  | Which surgical specialty is the study concerning? (if across more than one specialty please click all that apply) | Breast (includes axillary lymph nodes related to breast cancer)  Gynaecological (uterine, cervical, sentinel nodes related to gynaecological cancers)  Head/ neck (thyroid, oral)  Hepato-pancreatico-biliary (Liver, biliary system, pancreas)  Lower gastrointestinal (large bowel, rectum, anus)  Neurosurgery (Central Nervous System, brain, spinal cord)  Paediatric (brain tumors, neuroblastoma, rhabdomyosarcoma, Wilms' tumor, osteosarcoma etc)  Thoracic (lung)  Upper gastrointestinal (oesophageal/ stomach/ small bowel)  Urological (kidney, ureter, bladder, prostate, penile)  Other (please detail) |
| 2 |  | Do the authors state what type of study this is? | Yes  No |
| 2a | If yes, 2 | Please state here |  |
| 3 |  | Does this study report an IDEAL stage? | Yes  No |
| 3a | If yes, 3 | Please copy the authors' statement verbatim: |  |
| 4 |  | Is this study a case report? (Study containing one patient or study containing more than one patient all undergoing different interventions) | Yes  No |
| 5 |  | Is this study a case series? (A study containing more than one patient, all undergoing the same intervention in question) | Yes  No |
| 6 |  | Did this study include a comparison group? (e.g. No fluorescence, different dose of fluorescent agent, other demographic group)  **Please only include demographic comparison groups if these are clearly specified in advance (i.e. in the aim or methods. Please do not include subgroup analyses here).* | Yes  No |
| 6a | If yes, 6 | How many comparison arms? (excluding the intervention/ fluorescence group) | 1  2  3  4  5  >5 |
| 6b | Options according to 6a | Which category best describes comparison arm 1? | No fluorescence  Alternative dose of fluorescence agent  An alternative fluorescence agent/technique  The addition of a co-intervention (fluorescence element unchanged)  The comparison group was based on patient demographic/ selection |
|  | If other, 6a | Please describe here: |  |
| 6c |  | Does the paper describe a rationale for selecting the intervention used in comparison arm 1? | Yes  No |
| 6ci | If yes, 6c | Please record the statement verbatim here |  |
|  |  | [Repeat questions above for up to 3 comparison groups] I, ii, iii used for comparison arm 1-5 |  |
| 6d |  | Does the study report at the OUTSET how patients were selected to be in either the comparator or intervention group(s)? (NB: not applicable if the paper is reporting a RCT) |  |
|  |  | Please record the statement here, verbatim from the paper |  |
| 6e | If yes, 6 | Did the authors attempt to match patients in the comparison group? | Yes  No |
| 6f | If yes 6e | Please describe how patients were matched |  |
| 8 |  | Was the **study** prospective or retrospective?  *If the study states “this is retrospective review of prospectively collected data”—please record as retrospective.* | Prospectively  Retrospectively  Mixture of retrospective and prospective  Not reported |
| 9 |  | Is this a randomised study? |  |
| 10 |  | In the introduction or methods sections, did the paper include a clear aim for the work? | Yes  No |
| 10a | If yes, 10 | Select the most appropriate category for the study aim: (select one if possible) | - Testing for efficacy/ feasibility - Testing for safety/ adverse events - To gain FDA/NICE/other approval - Describing the technique - Optimising the technique - Evaluating the learning curve - "Describing our centre's experience" - Comparing patient outcomes - Other |
| 10b | If yes, 10 | Record the statement here verbatim from the paper |  |
| 11 |  | Is the **actual** follow-up period reported?  *Please note that this is different to the intended follow up period or method of follow up.* | Yes  No |
| 11a |  | Enter average follow-up time in months (If reported) |  |
| 11b |  | Enter follow-up range in months (If reported) |  |
| 12 |  | Does the study report prior publication of a protocol? | Yes  No |
| 12a | If yes, 12 | Has the protocol’s citation been provided? | Yes  No |
| 12b | If yes, 12a | Please provide citation/link. |  |
| **C1:** **Centre Details** | | | |
| 1 |  | Number of centres  *Please only record if explicitly stated in the paper* (i.e. do not use information in the "author affiliations" section) | Single centre  Multi centre  Not reported |
| 1a | If multi,1 | If multicentre, how many centres?  *Please only record if explicitly stated in the paper* |  |
| 2 |  | Country of study  *Please check author affiliations for this question* | Multiple  [Full list of countries] |
| 2a |  | If multiple, please list |  |
| 3 |  | Type of centre  *Please only record if explicitly stated in the paper* | General/secondary care  Specialist/regional/supraregional/tertiary/ University  Mixed (if multicentre)  Children’s Hospital  Not specified |
| 3a | If mixed, 3 | If multicentre, please record the proportion of each centre type here |  |
| 4 |  | Institution name(s)  *Please check author affiliations for this question* |  |
| 5 |  | Study period in which procedures were performed |  |
| 6 |  | Is the usual case load of the surgical procedure of interest reported? e.g. number of cases per year or within a defined period | Yes  No |
| 6a | If yes, 6 | Please copy verbatim |  |
| 7 |  | Is the usual case load of the surgical procedure of interest WITH FLUORESCENCE reported? e.g. number of cases per year or within a defined period |  |
| 7a | If yes, 7 | Please copy verbatim |  |
| **Section C2: Data about the operating surgeons and quality assurance** | | | |
| 1 |  | Is the number of surgeons that performed the surgical procedure reported? | Yes  No |
|  | If yes, 1 | Please record verbatim |  |
| 2 |  | Is there a statement reporting the grade of the surgeon(s) in the study? | Yes  No |
| 2a | If yes, 2 | What is the grade(s) of the participating surgeons? | Consultant/ attending  Trainee/ resident/ fellow  Mixed consultant and trainee  Not reported |
|  | If yes, 2 | Please record verbatim |  |
| 3 |  | Did the authors state any pre-specified criteria for surgeons to be eligible to participate in this study? (for example, does the paper state that participating surgeons must have used fluorescence guided surgery a predetermined number of times?) | Yes  No |
| 3a | If yes, 3 | What were these criteria and how (if at all) were they justified? (record here, verbatim from paper): |  |
| 4 |  | Is there a statement outlining what training surgeons received prior to their first in-human procedure? | Yes  No |
| 4a | If yes, 4 | What surgeon training was provided prior to their first in-human procedure? | Human cadaver  Virtual simulation  Simulation using models  Observation  Animal (alive)  Animal (cadaveric)  Accredited course  Other |
| 4b | If yes, 4 | Please record verbatim |  |
| 5 |  | Is the previous number of the surgical procedure of interest WITH fluorescence performed by the surgeon(s) reported? |  |
| 5a | If yes, 5 | Please record verbatim |  |
| 6 | If yes,6 | Is the previous number of the surgical procedure of interest WITHOUT fluorescence by surgeon(s) reported? |  |
| 6a | If yes, 6 | Please record verbatim |  |
| 7 |  | Is the previous number of other surgical procedures WITH fluorescence performed by surgeon(s) reported? |  |
| 7a | If yes, 7 | Please record verbatim |  |
| 8 |  | Do the authors include a generic statement of surgeon experience? | Yes  No |
| 8a | If yes, 8 | Please record verbatim |  |
| 9 |  | Does the study assess/report the learning curve in relation to intra-operative fluorescence imaging? | Yes  No |
| 9a | If yes, 9 | If Yes, please provide a verbatim description of how it was assessed |  |
| 9b | If yes, 9 | Was the learning curve discussed as a limitation or advantage of intra-operative fluorescence imaging? | The learning curve was reported as a DISADVANTAGE of intra-operative fluorescence imaging.  The learning curve was reported as an ADVANTAGE of intra-operative fluorescence imaging.  No. The learning curve was not reported as an advantage OR disadvantage of intra-operative fluorescence imaging. |
|  | If adv or disadv 9b | Please record verbatim |  |
| 10 |  | Were any of the following methods of supporting surgeon performance reported in the study? | Proctorship or mentorship  Independent committee oversight  Dual consultant/ surgeon operating  Other |
| 10a | If yes, to any of 10 | Please provide details of methods of supporting surgeon performance Please provide for all of those answered "yes" above |  |
| 11 |  | Were any of the following strategies for monitoring standards of surgery reported in the study?  (Select those that apply. These do not include photographs included in the manuscript used to demonstrate technique) | Information recorded in case report forms (CRFs)  Photographic or video evidence used to record the completion of key operative steps  Independent observation of surgical techniques during the study (e.g. mentoring)?  Other |
| 11a | If yes to any of 11 | Please provide details of methods of monitoring standards of surgery Please provide for all of those answered "yes" above |  |
| Section D1: Data about the surgical procedure | | | |
| 1 |  | Please record the name of surgical procedure(s) verbatim e.g. anterior resection, lung metastasectomy, radical prostatectomy, sentinel node biopsy for breast cancer etc. |  |
| 2 |  | What was the surgical access of the procedure? | Open  Laparoscopic  Robotic  Other |
| 2a |  | If other, please record further details |  |
| Section D1: Data about the patient participants | | | |
| 1 |  | Total number of patients in the study  *If the study reports data for both the total cohort and a smaller cohort of matched patients, please include the total cohort here. Then please use the speech bubble icon to add a comment stating the matched cohort sizes.* |  |
| 2 |  | Number of participants in the intervention group (fluorescence guided surgery)  *See comment on Q1 for matched studies.* |  |
| 2 | Repeated depending on B5a | Number of participants in the comparison group [1-5]. Please use the same group numbers that you allocated in Form B |  |
| 3 |  | Average age of patients in intervention (fluorescence guided surgery) group  *If the study reports data for both the total cohort and a smaller cohort of matched patients, please record both cohorts here with square brackets to clarify group e.g. [Total cohort] Mean age 45 [Matched cohort] Mean age 39.* |  |
| 3 | Repeated depending on B5a | Average age of patients in comparison group [1-3] |  |
| 4 |  | Sex (% of males) in intervention (fluorescence guided surgery) group  *See Q4 comment for matched studies* |  |
| 4 | Repeated depending on B5a | Sex (% of males) in comparison group [1-3] |  |
| 5 |  | American Society of Anesthesiologists (ASA) grades in intervention (fluorescence guided surgery) group  *See Q4 comment for matched studies* |  |
| 5 | Repeated depending on B5a | American Society of Anesthesiologists (ASA) grades in comparison group [1-3] |  |
| 6 |  | Average BMI in intervention (fluorescence guided surgery) group  *Please record average and range if recorded. See Q4 comment for matched studies* |  |
| 6 | Repeated depending on B5a | BMI in comparison group [1-3] |  |
| 7 |  | Indication for surgery in the intervention (fluorescence) group  *e.g. colonic cancer resection. Please record as %. See Q4 comment for matched studies* |  |
| 7 | Repeated depending on B5a | Indication for surgery in comparison group [1-3]  *e.g. colonic cancer resection. Please record as %.* |  |
| 8 |  | **Overall cancer stage in the intervention group**  Please record as % e.g. I = 20%, II = 40%, III = 40%  State "NR" if not available |  |
| 8 | Repeated depending on B5a | **Overall cancer stage in the comparison group**  Please record as % e.g. I = 20%, II = 40%, III = 40%  State "NR" if not available |  |
| 9 |  | **Please record the CLINICAL/RADIOLOGICAL tumour (T stage) of the intervention group**  *Do not record the pathological T stage here. Please specify whether clinical or radiological stage was provided.*  *Please record as %. e.g. T1 = 40%, T2 = 60%*  *See Q4 comment for matched studies* |  |
| 9 | Repeated depending on B5a | **Please record the CLINICAL/RADIOLOGICAL tumour (T stage) of the comparison group**  *Do not record the pathological T stage here. Please specify whether clinical or radiological stage was provided.*  *Please record as %. e.g. T1 = 40%, T2 = 60%*  *See Q4 comment for matched studies* |  |
| 10 |  | **Please record the CLINICAL/RADIOLOGICAL nodal (N) stage of the intervention group**  *Do not record the pathological N stage here. Please specify whether clinical or radiological stage was provided.*  *Please record as %. e.g. N0 = 40%, N1 = 60%*  *See Q4 comment for matched studies* |  |
| 10 | Repeated depending on B5a | **Please record the CLINICAL/RADIOLOGICAL nodal (N) stage of the comparison group**  *Do not record the pathological N stage here. Please specify whether clinical or radiological stage was provided.*  *Please record as %. e.g. N0 = 40%, N1 = 60%*  *See Q4 comment for matched studies* |  |
| 11 |  | Other patient demographics reported in paper but not extracted |  |
| 12 |  | Please record any significant differences in baseline demographics between intervention and comparison group(s) |  |
| 13 |  | Did the authors include a statement about inclusion and exclusion criteria for patients in the abstract, introduction or methods?  *If a study reports indications for surgery* *only, then please select “no”* | Yes  No |
| 13a | If yes, 13 | Inclusion criteria |  |
| 13b | If yes, 13 | Exclusion criteria |  |
| 14 |  | Did the authors report any modifications to which patients were offered the procedure DURING the study | Yes  No |
| 14a | If yes, 14 | Record verbatim |  |
| 15 |  | Did the authors include a statement about what happened to patients who did not meet the inclusion criteria to receive the intervention (or to participate if it was a comparative study)? | Yes  No |
| 15a | If yes, 15 | Please record the statement here, verbatim from the paper |  |
| 16 |  | Do the authors state that the study included consecutive patients? | Yes  No |
| 16a | If yes, 16 | Please record statement here verbatim |  |
| Section E: Data about governance and ethical factors | | | |
| 1 |  | Was there a statement confirming IRB*/ethics committee approval? (* Institutional Review Board) | Yes  No |
| 1a | If yes, 1 | Please enter statement confirming IRB/ ethics committee approval verbatim including any IRB/ethics approval number : |  |
| 2 |  | Was there a statement confirming individual patient consent? | Yes  No |
| 2a | If yes, 2 | Record consent statements here (verbatim from paper) including whether this consent was to the innovative procedure OR to the research project OR both |  |
| 3 |  | Was information provided about FDA/NICE/CE marking/clinical effectiveness approval? | Yes  No |
| 3a |  | Record the statement here, including whether that approval was for the device in general (e.g. NIR fluorescence) or for a specific clinical indication (e.g. anastomotic perfusion) |  |
| 4 |  | Was there a statement that patients were specifically informed about the innovative nature of the intervention? | Yes  No |
| 4a | If yes, 4 | Record this statement here (verbatim from paper) |  |
| 4b | If yes, 4 | Who gave this information to the patients? (e.g. surgeon, nurse, other) |  |
| 5 |  | Did the paper report any amendment to the IRB/ethics approval AFTER the study had started? | Yes  No |
| 5a | If yes, 5 | Include any statement here (including if the amendment was rejected, why the amendment was sought) |  |
| 6 |  | Does this study claim to introduce an entirely **new** intervention or **new** modification of an existing intervention? | Yes  No |
| 6a | If yes, 6 | Was there a statement confirming that the participant(s) were told this? | Yes  No |
| 6b | If yes, 6a | Record that statement here, verbatim from paper |  |
| 7 |  | Was information provided about the number of patients declining the intervention? | Yes  No |
| 7a | If yes, 7 | If yes, record this information here (verbatim from paper) (NB: this is different from the number of patients found to be ineligible for the intervention) |  |
| 8 |  | Did the authors describe additional sources of information about the intervention(s) provided for patients such as information leaflets, web pages or other sources? | Yes  No |
| 8a | If yes, 8 | What were these sources? |  |
| 8b | If yes, 8 | Were links or references to these sources included in the paper? | Yes  No |
| 8c | If yes, 8 | Please provide links or describe these sources below. |  |
| 9 |  | Does the study report prior registration with a trials register, such as clinicaltrials.gov, ISRCTN or the Cochrane Central Register of Controlled Trials (CENTRAL)?  Note that individual hospital databases do not count here. | Yes  No |
| 9a |  | Which database? |  |
| 9b |  | What is the registration identification code? |  |
| **Section F: Data about** **modifications made to the intervention and/or co-intervention** | | | |
| 1 |  | Does the study report at the OUTSET that it was of a modification/ refinement/ adaptation of fluorescence guided surgery? | Yes  No |
| 1a | If yes, 1 | What was the nature of the modification/refinement/adaptation? | Use of fluorescence guided surgery for a new disease indication  Use of fluorescence guided surgery for as a new element of an existing operation  Use of fluorescence guided surgery in a new patient group  Modification of fluorescence technique (e.g. Describing a new way for surgeons to use the fluorescence, quantification of fluorescence)  Other |
| 1b | If yes, 1 | Briefly describe the modification here |  |
| 1c | If yes, 1 | Was the rationale/justification for the modification described? | Yes  No |
| 1d | If yes, 1c | Briefly describe that rationale/justification here |  |
| 2 |  | Does the study report any modifications/refinement/adaptation to fluorescence guided surgery that took place AFTER the study had started?  Including any of the technical steps. | Yes  No |
| 2a | If yes, 2 | What was the nature of the modification/refinement/adaptation? | Use of fluorescence guided surgery for a new disease indication  Use of fluorescence guided surgery for as a new element of an existing operation  Use of fluorescence guided surgery in a new patient group  Modification of fluorescence technique (e.g. Describing a new way for surgeons to use the fluorescence, quantification of fluorescence)  Other |
| 2b | If yes, 2 | Briefly describe the modification here |  |
| 2c | If yes, 2 | Was the rationale/justification for the modification described? | Yes  No |
| 2d | If yes, 2c | Briefly describe that rationale/justification here |  |
| 3 |  | Did the authors report any modifications to any of the accompanying interventions (i.e. co-interventions)? | Yes  No |
| 3a | If yes, 3 | Briefly describe the modification here |  |
| 3b | If yes, 3 | Was the rationale/justification for the modification described? | Yes  No |
| 3c | If yes, 3b | Briefly describe that rationale/justification here |  |
| 4 |  | If there WAS a modification, is it reported that patients were told about this? | Yes  No  Not applicable (no modification) |
| 4a | If yes, 4 | Record the statement here, verbatim from paper (including any detail about how this information was given - e.g. leaflets) |  |
| Section G: Data about the intervention | | | |
| 1 |  | What was the purpose of the use of NIR fluorescence guided surgery? | Sentinel lymph node mapping  Specific tumour visualization (ie. Highlighting tumour by binding to specific markers on tumour surface) Aspecific tumour visualization (ie. fluorescence based on tumour blood supply)  Blood supply around tumour (to reduce damages to surrounding vascular structures)  Blood supply to tumour (to guide vessel clamping)  Fluorescence endoscopy  Vascularisation of tissue for reconstruction (e.g. skin flap, colonic anastomosis)  Other |
| 1a |  | Please copy verbatim |  |
| 2 |  | Do the authors reference the fluorescence technique of a previous study in their methods section? | Yes  No |
| 2a | If yes, 2 | Please record verbatim |  |
| 2b | If yes, 2 | Please copy the study referenced here: |  |
| 3 |  | Which fluorescence agent was used? | Indocyanine green (ICG)  Methylene Blue (MB)  IRDye800CW  Other, please specify |
| 4 |  | Was the contrast agent labelled with another substance (e.g. antibody, nanoparticle)? | Antibody  Nanoparticle  Nanocolloid  Radiolabeled substance  Other  None |
| 4a | If yes, 4 | Please record verbatim |  |
| 5 |  | Do the authors report how the fluorescence agent was constituted (e.g. the vial of ICG powder was reconstituted at the concentration of 25 mg/10 mL) | Yes  No |
| 5a | If yes, 5 | Please record verbatim |  |
| 6 |  | Do the authors state at how many points the fluorescence agent was administered? | Not stated  1  2  3  4  Other |
| 7 |  | Please record verbatim at which time point the first dose of fluorescence agent was administered (e.g. 24 hours prior to surgery, after decision on proximal site of division, after tumour mobilisation prior to lymph node dissection, after anaesthetic but prior to knife to skin) |  |
| 7b |  | What dose of fluorescence agent was given in the first administration? |  |
| 7c |  | What was the mode of administration of fluorescence? | Intravenous  Oral  Directly into tissue (if so, which) |
|  | Repeat 7a, b & b according to question 6 |  |  |
| 7 |  | Do the authors report the model/type of near-infrared imaging system used in the study? (i.e. Hamamatsu Photonics, Karl Storz, Stryker, MizuhoMedical Co, Novadaq Technologies, in house build) | Yes  No |
| 7a | If yes, 7 | Please record here verbatim |  |
| 7b |  | Do the authors report any modifications to the imaging system? |  |
| 7c |  | Do the authors report the light source used | Yes  No |
| 7d |  | Please record here verbatim |  |
| 7e |  | Do the authors report the filtering used | Yes  No |
| 7f |  | Please record here verbatim |  |
| 8 |  | Was fluorescence imaging used in real-time to guide the surgery? | Yes  No |
| 9 |  | What type of display was used intra-operatively? | Standard screen  3D screen  Immersive, e.g. da Vinci console  Other, e.g. head-mounted  Not stated |
|  |  | Please record verbatim |  |
| 10 |  | Was fluorescence provided as an overlay (combined display of white light and fluorescence)? | Yes  No  Not stated |
|  |  | Please record verbatim |  |
| 12 |  | Was the intra-operative visual data saved/ stored? | Yes  No  Not stated |
|  |  | Please record details verbatim |  |
|  | If yes, 12 | Is the stored data available in an online repository? | Yes  No |
|  |  | Please record details verbatim |  |
| 12a | If yes, 12 | Has the stored data been used for quantitative analysis? | Yes  No |
|  |  | Please record details verbatim |  |
| 12b | If yes, 12 | Has the data been labelled or annotated? | Yes  No |
|  |  | Please record details verbatim |  |
|  | If yes, 12b | How many researchers performed labelling? | 1  2  3  4 |
| 13 |  | Are the labels and data available for machine learning or other computational analysis research? | Yes  No |
|  | If yes, 13 | If yes, please record verbatim |  |
| Section K1: Outcomes: COHESIVE | | | |
| 1 |  | Did the authors state the overall desired effect (overall aim) of THE PROCEDURE?  Note that this is different to the aim of the study. e.g. "the objective of the procedure was to remove the cancer" | Yes  No |
| 1a | If yes, 1 | Please record verbatim |  |
| 1b | If yes, 1 | Did the authors report the number of patients in whom the overall desired effect (overall aim) of the procedure was achieved?  e.g. the number of patients in whom the cancer was completed resected | Yes  No |
| 1c | If yes, 1b | Please record verbatim |  |
| 2 |  | Did the authors report the number of patients in whom the planned procedure was completed, either with/without modifications? | Yes  No |
| 2a | If yes, 2 | Enter text verbatim |  |
| 3 |  | Did the authors report the number of patients in whom the planned procedure was abandoned or changed to another procedure? | Yes  No |
| 3a | If yes, 3 | Enter text verbatim |  |
| 4 |  | Did the authors report any problems with the specialised equipment? (e.g. imaging system malfunctioned) | Yes  No  Not applicable |
| 4a | If yes, 4 | Please record details here verbatim |  |
| 4b |  | Did the authors report the number of patients in whom the specialised equipment did/did not work? | Yes  No  Not applicable |
| 4c | If yes, 4b | Please record details here verbatim |  |
| 5 |  | Did the authors state any intended benefits of the procedure?  NB: this includes benefits occurring before, during or after the procedure | Yes  No |
| 5a | If yes, 5 | Were the stated benefits of the procedure…  Tick as many as apply: | Before (e.g. fewer tests needed before surgery).  During (e.g. less operative time).  After (e.g. fewer complications). |
| 5b | If yes, 5 | Please provide details verbatim. |  |
| 6 |  | Did the authors state any expected disadvantages of the procedure?  e.g. longer operative time, more equipment or theatre staff needed. NB: this includes disadvantages occurring before, during or after the procedure. |  |
| 6a | If yes, 6 | Was the stated expected disadvantage of the procedure…  Select as many as apply | Before (e.g. more tests needed before surgery).  During (e.g. longer operative time).  After (e.g. more patient required intensive care). |
| 6b | If yes, 6 | Please provide details verbatim. |  |
| 7 |  | Did the authors report any unexpected disadvantages or problems with the new procedure  e.g. unexpected complication, | Yes  No |
| 7a | If yes, 7 | Please provide details verbatim |  |
| 8 |  | Did the authors report any details about surgeons’ experience of the procedure?  NB: this includes emotional, psychological and physical experiences (e.g. comfort, stress, difficulty). | Yes  No |
| 8a | If yes, 8 | Please provide details verbatim |  |
| 9 |  | Did the authors report any details about patients’ experience relating to the procedure being innovative?  NB: this includes emotional, psychological and physical experiences (e.g. anxiety because of the procedure being new). | Yes  No |
| 9a | If yes, 9 | Please provide details verbatim |  |
| 1 |  | Does the study report using a Core Outcomes Set? | Yes  No |
| 1a | If yes 1 | If yes, has a citation been provided? |  |
| 1b | If yes 1 | If yes, record citation verbatim. |  |
|  |  | Outcome name  *Please record verbatim* |  |
| a |  | Is the outcome introduced in the abstract, introduction or methods? | Yes  No |
| b |  | Is the result of the outcome reported? | Yes  No |
| c |  | Is the outcome defined? | Yes  No |
|  | If yes, c | Please copy the definition used for this outcome verbatim |  |
| ci | If yes c | Is a citation for the definition provided? | Yes  No |
|  | If yes, ci | Citation for definition |  |
| d |  | Has its method of measurement been described? | Yes  No |
|  | If yes d | If yes, record description of method of measurement verbatim. |  |
| di | If yes d | If yes, has the method been developed for this study, or did it originate elsewhere? | Developed for this study  Originated elsewhere  Not reported |
|  | If originated elsewhere | If originated elsewhere, is there a citation for the original report of the method? |  |
| 6d | If yes, 6c | If yes, record citation verbatim. |  |
| 7 |  | Does the study report who the assessors of the outcome were? | Yes  No |
| 7b | If yes, 7 | If yes, record who assessed verbatim. |  |
| 8 |  | Is this a primary or secondary outcome? | Primary  Secondary  Not reported |
| 9 |  | Did the authors state at which point the outcome was measured? | Yes  No |
| 9a |  | If yes, | **Dropdown:**  Intraoperative  Inpatient (including length of stay)  30 days  3 months  6 months  1 year  2 years  3 years  4 years  5 years  “At last follow up”  MULTIPLE  Other |
| 9b | If other | Please record verbatim |  |
| 9c | If multiple | Please record verbatim |  |
| 10 |  | What are the units of measurement? For non-numerical outcomes, please state whether the outcome is reported as a percentage, proportion, ratio or statement |  |
| **Section K2: Outcome Reporting** | | | |
| 1 |  | Does the study report using a Core Outcome Set | Yes  No |
|  |  | If yes, please provide citation |  |
| 2 |  | Outcome name |  |
| 2a |  | Is the outcome introduced in the abstract, introduction or methods sections? | Yes  No |
| 2b |  | Is the result of the outcome  reported? | Yes  No |
| 2c |  | Is the outcome defined | Yes  No |
| 2d |  | Is a citation for the definition provided | Yes  No |
| 2e |  | Has its method of measurement been described | Yes  No |
| 2f |  | Does the study report who the assessors of the outcome were | Yes  No |
| 2g |  | Did the authors state at which time point the outcome was measured? | Yes  No |
| 2h |  | Is this a primary or a secondary outcome | Primary  Secondary  Not specified |
|  |  | Repeat 2-2h for each outcome reported |  |
| **Section M: Summing up. Considering the DISCUSSION and CONCLUSION of the paper** | | | |
| 1 |  | Select one of the following statements to most closely reflect the authors' conclusion about near-infrared fluorescence guided oncological surgery from the findings of their study  Select one if possible | [Have made multiple selection possible as often multiple conclusions]  The authors recommend further INNOVATION (or development/ refinement/ adaptation) of fluorescence guided oncological surgery is required.  The authors recommend further EVALUATION of fluorescence guided oncological surgery is required (e.g. they recommend evaluation in an RCT).  The authors recommend ADOPTION of fluorescence guided oncological surgery into routine clinical practice.  The authors recommend STOPPING the use of fluorescence guided oncological surgery in routine clinical practice.  The author's conclusions relate to prediction of patient outcomes.  The authors make a different conclusion to those above. |
| 1a |  | Summarise/ record their conclusion here: |  |
